# Impact of clinical pharmacist interventions on medication administration via enteral feeding tubes in a neurology ward: a pre- and post-educational prospective study

**DOI:** 10.3389/fphar.2025.1519835

**Published:** 2025-04-10

**Authors:** Yunus Emre Ayhan, Ömer Faruk Özkanlı, Şeyma Gözelizmir, Anmar Al-Taie, Mesut Sancar, Ipek Midi

**Affiliations:** ^1^ Clinical Pharmacy Department, Prof. Dr. Cemil Taşcıoğlu City Hospital, Istanbul, Türkiye; ^2^ Clinical Pharmacy Department, Faculty of Pharmacy, Marmara University, Istanbul, Türkiye; ^3^ Clinical Pharmacy Department, Faculty of Pharmacy, Istinye University, Istanbul, Türkiye; ^4^ Department of Neurology, Faculty of Medicine, Marmara University, Istanbul, Türkiye

**Keywords:** clinical pharmacist, drug administration, enteral feeding tube, neurology, modified released dosage forms

## Abstract

**Objectives:**

The aim of this study was to evaluate the effects of clinical pharmacists’ education and interventions on the appropriateness of dosage forms of drugs administered via the enteral feeding tubes (EFTs) in hospitalised patients in the neurology ward.

**Methods:**

This was a prospective, pre-post intervention study conducted among patients and neurologist team professionals in the neurology ward of a training and research hospital in Istanbul, Türkiye. The study was designed in two phases as a pre-education observation period (OP) and a post-education intervention period (IP), during which the clinical pharmacists provided the required recommendations. Medications evaluated in terms of EFT-related medication administration errors (EFTRMAE) during the hospitalisation and discharge of patients in OP and IP. The knowledge levels of the neurologist team regarding EFT medication administration were collected and evaluated with an online survey before and after the education program.

**Results:**

A total of 68 patients were included in the study, with 34 patients in the OP and 34 in the IP. During hospitalisation, EFTRMAEs were observed in 24 patients (70.6%) in the OP, whereas in the IP, EFTRMAEs were detected in 13 patients (38.2%) before clinical pharmacist interventions (p = 0.014). Throughout hospitalisation in the IP group, clinical pharmacists provided 25 interventions related to EFTRMAEs, of which 84% were accepted by physicians. However, only 11 of the accepted recommendations were fully implemented. Following these interventions, inappropriate drug administration via EFT remained in only 5 patients (14.7%) (p < 0.001). At hospital discharge, the EFTRMAE rate, which was 76.5% in the OP group, decreased to 23.5% in the IP group (p < 0.001). The neurologist team’s knowledge of EFT medication administration improved significantly following clinical pharmacist education, with the average number of correct responses increasing from 16.1 ± 4 before the education to 21.1 ± 2.1 afterward (p < 0.001).

**Conclusion:**

EFTRMAEs are frequently encountered in patients hospitalised in the neurology ward. Including clinical pharmacists in the healthcare team and the education program provided to physicians and nurses will increase the knowledge level of participants and the ability of physicians to prescribe appropriate dosage forms for administration via EFT.

## Introduction

Patients suffering from neurological conditions frequently struggle with malnutrition, which can decrease mobility, and other comorbidities that impact metabolic function (Nieuwenhuizen et al., 2010; Sheard, 2014). As a result, the nutritional management is a key component of the multidisciplinary care given to patients with neurological conditions. On the other hand, dysphagia can impact the pharmacological management of patients with such disorders since it causes problems with the oral administration of medications, which can worsen the disease itself (Nieuwenhuizen et al., 2010). Therefore, such patients require artificial nutrition support by parenteral or enteral feeding tube (EFT) when symptoms of disease severely impede oral intake (Stavroulakis and McDermott, 2016). In many hospitalised patients, when liquid, transdermal, rectal, and intravenous formulations are not accessible or preferred, medications are often administered through EFTs (Williams, 2008). It is also noteworthy that many dosage forms developed to simplify patients’ dosing regimens and improve treatment adherence, such as sustained-release, modified-release formulations, and enteric-coated (EC), have been reported to be inappropriate for administration via EFT (Buhrer Ferreira Neto et al., 2016; Williams, 2008).

Several earlier studies have been carried out to reduce EFT-related medication administration errors (EFTRMAE) through diverse methods of interventions, such as education under the management of nurses, daily visits of pharmacist technicians, creating alarms in medication order systems, placing warning symbols on the medications to be administered, and using clinical decision support systems (Wasylewicz et al., 2021)^.^ Thus, the challenge that medical practitioners face every day is selecting formulations whose alteration does not change pharmacological efficacy in order to prevent unwanted therapeutic failures.

Clinical pharmacists are well-known for their critical role in the multidisciplinary healthcare teams. They can play an important role in reducing health disparities by providing patient education and counselling, medication adherence support, monitoring, and providing knowledge on drug-related problems (DRPs) (Cengiz, Midi, and Sancar, 2025). As a result, interprofessional collaboration is essential to enhance patient’s quality of life (Al-Taie, 2023). However, scarce studies were conducted by clinical pharmacists to increase the knowledge level of healthcare professionals regarding the appropriateness of dosage forms and administration methods of drugs administered via EFT. These studies demonstrated that educational interventions provided by pharmacists are effective in increasing healthcare professionals’ knowledge of medication administration and decreasing medication errors via EFTs (Abu Hdaib, Albsoul-Younes, and Wazaify, 2021; Alhashemi, Ghorbani, and Vazin, 2019; Dashti-Khavidaki et al., 2012; Hanssens et al., 2006; Idzinga, De Jong, and Van den Bemt, 2009; Lohmann et al., 2015; Zuccari et al., 2022). From this perspective, the aim of this study was to evaluate the effects of clinical pharmacists’ education and interventions on the appropriateness of dosage forms and administration methods of drugs administered with EFT in patients hospitalised in the neurology ward in Istanbul, Türkiye.

## Materials and methods

### Study design and participants

This was a prospective, pre-post intervention study conducted among patients and neurologist team professionals in the neurology ward of a training and research hospital in Istanbul, Türkiye. The study was designed in two phases between February 2023 and March 2024 for a total study duration of 13 months. The first part consisted of a pre-education observation period (OP) and a post-education intervention period (IP), during which the clinical pharmacists provided the required recommendations. Patients hospitalised in the neurology ward for ≥24 h who were ≥18 years old and who had at least one drug administered via EFT were included in the study. Patients with missing data and those lost to follow-up were excluded from the study. Exclusion criteria also included records lacking essential information, such as medication administration details via EFT or follow-up documentation. Rather than requiring a specific minimum follow-up duration, the focus was on ensuring the availability of complete data on medication administration and medication errors during hospitalization and at discharge.

Regarding the sample size, the calculation was made on alpha 0.05% and 95% power values, and considering the waste margin of 15%, it was decided to include at least 40 patients (Van Welie et al., 2016).

During the OP, clinical pharmacists did not provide any interventions or recommendations; they solely observed and documented medication administration errors via EFT. In contrast, during the IP, following the education program, clinical pharmacists actively provided interventions to optimize medication administration.

### Evaluation of drug administration through enteral feeding tubes and clinical pharmacist interventions

The design of the OP was to solely observe and record the administration of drugs via EFT, without any recommendations or interventions. The OP lasted for 6 months, during which drug treatment for the patients received during their stay in the neurology ward and the medications prescribed at the time of the patient’s discharge were recorded. The second IP was designed to be an educational part during which a face-to-face education was organised for 2 h every week and lasted 1 month with the neurologist team professionals in the neurology ward. The clinical pharmacists and neurologist team professionals worked collaboratively during both the admission and discharge of patients, thereby optimising the patient’s treatment. This collaboration included evaluating the appropriateness of the medications administered via the EFT, use of appropriate dosage forms, removal of inappropriate medications administered via the EFT, recommendations to use an alternative route of administration, and optimising the application procedure. Meanwhile, during the IP, the number and type of recommendations provided to the neurologist team professionals and the rate of recommendation acceptance were recorded.

The education was designed to be highly practical, with hands-on exercises and real-life case studies to ensure the participants were fully prepared and capable. The content of the education has been prepared to include drug administration processes through EFTs, introduction of drug dosage forms, inappropriate drug administration frequently given during the OP, and suggestions to solve and correct drug administration errors. In the case of EFTRMAE detection, the clinical pharmacist presented his recommendations to the patient’s primary physician to ensure the prescription of appropriate drug dosage forms via the EFT.

**FIGURE 1:**
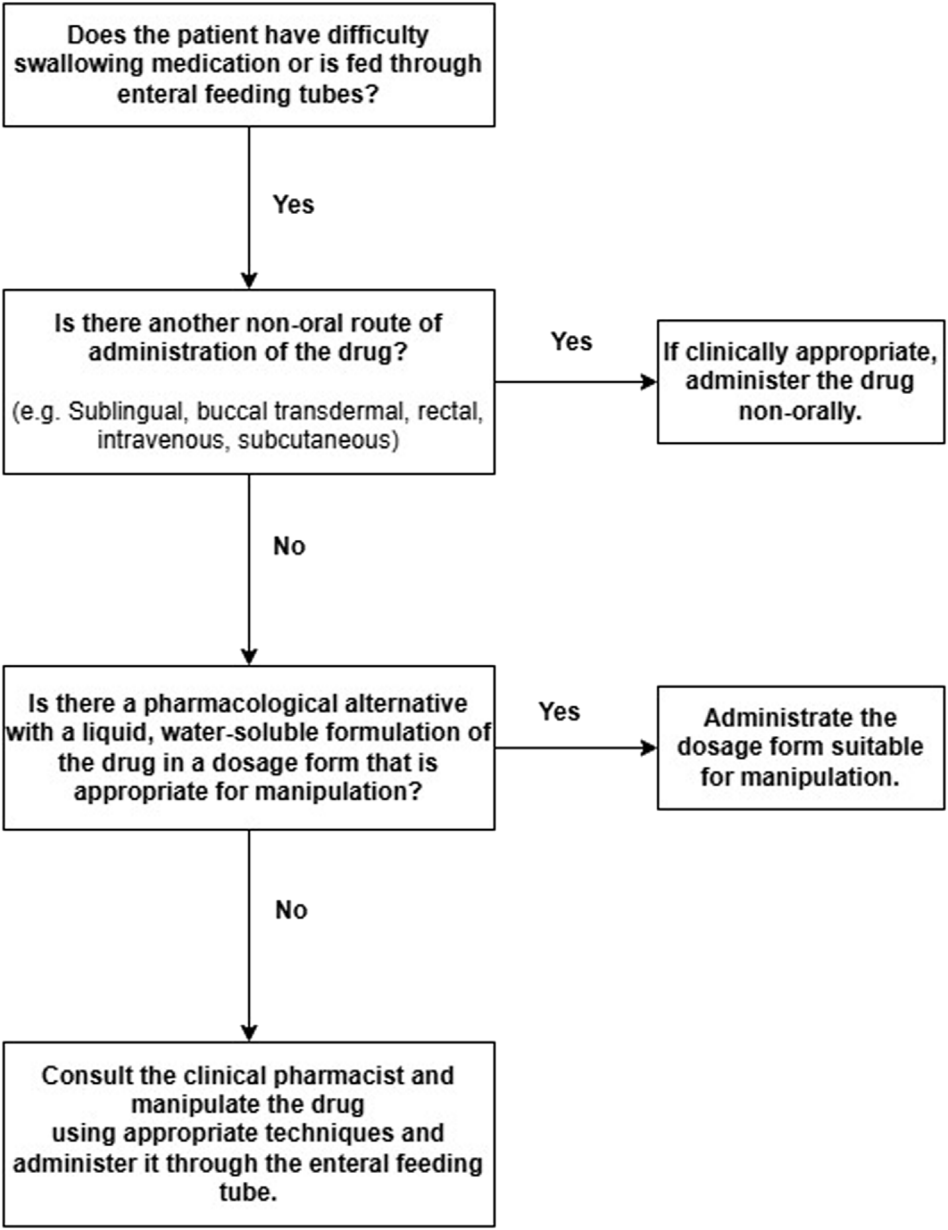
Flowchart for evaluating drug administration through enteral feeding tubes.

The clinical pharmacist’s recommendations also included discontinuing inappropriate medications via the EFT and manipulating appropriate and alternative medications when the drug dosage form was unavailable. The acceptance and implementation of the clinical pharmacist’s recommendations were recorded. Figure 1 presents a flowchart for evaluating drug administration through EFTs in this study.

### Study sources and definitions

The appropriateness of drug administration through EFT was evaluated using ASPEN Safe Practices for Enteral Nutrition Therapy and Handbook of Drug Administration via Enteral Feeding Tubes, Third Edition (Bankhead et al., 2009; White and Bradnam, 2018). In addition, we used UpToDate^®^ (Wolters Kluwer Health Inc., 2023) reference sources as resources to support our evidence-based clinical decision-making. We also utilised the Türkiye Medicines and Medical Devices Agency Brief Product Information/Instructions for Use and local resources of Rx Media Pharma^®^ Interactive Drug Information Source to recommend drug dosage forms available in Türkiye for the application of drugs and to screen different dosage forms.

The educational content was prepared based on ASPEN Safe Practices for Enteral Nutrition Therapy and Handbook of Drug Administration via Enteral Feeding Tubes, Third Edition, and UpToDate^®^ (Wolters Kluwer Health Inc., 2023), which were also the primary sources used to assess medication appropriateness for EFT administration (Bankhead et al., 2009; White and Bradnam, 2018).

Manipulation has been defined as “all activities performed prior to administration so that the drug can be administered to the patient using an alternative strategy (e.g., to increase patient acceptability or adjust the dose)” (Zahn et al., 2020). Administration of medications with EFT by manipulating dosage forms that were not appropriate for administration with EFT in terms of the dosage form or that were not used on the patient despite having appropriate preparations on the market and that should not be broken or crushed was considered EFTRMAE. Possible inappropriate practices regarding nursing services were not taken into account when administering medications via EFT. Only the applicability of drugs and dosage forms from EFT was taken into account. The flow chart in Figure 1 evaluated the appropriateness of drugs to be administered with EFT and recommended alternative dosage forms.

### Data collection and outcomes

Data were recorded from patients’ files, medication orders, the hospital information management system (HIMS), laboratory results, and treatment details, including medication use, dosage forms, administration methods, discharge prescriptions, and disease-related scores while ensuring compliance with personal confidentiality conditions. The HIMS, an electronic medical record system, was used to document patient demographics, laboratory results, and medication orders. The Functional Oral Intake Scale (FOIS) was used to assess the range of oral intake in patients. During this study, FOIS was applied which is a measure with high reliability, validity, and sensitivity to change for determining and monitoring the oral intake range of patients (Crary et al., 2005). The seven-tiered ordinal scale, a comprehensive tool for analysing oral food and beverage consumption, encompasses a wide range of feeding options. Level 1 signifies “nothing by mouth,” Level 2 indicates “tube dependent with minimal attempts of food or liquids,” Level 3 denotes “tube dependent with consistent oral intake of food or liquids,” Levels 4–6 represent “expansion of oral diet not reached,” and Level 7 is defined as “expansion of oral diet reached” (Coppens et al., 2016).

An online questionnaire was administered to assess the knowledge levels and attitudes regarding the appropriateness of drug dosage forms and administration methods via EFTs among neurologist team professionals during both pre- and post-education phases. The self-developed instrument was constructed based on an extensive literature review and tailored to the study’s objectives. Each item was rigorously evaluated by a panel of clinical pharmacists and neurologists for relevance, clarity, and comprehensiveness, and a forward-backward translation procedure was implemented to ensure both linguistic and cultural appropriateness, thereby affirming the instrument’s content validity. To further assess the instrument’s reliability and clarity, a pilot test-retest study was conducted with 10 neurology team professionals from outside the institution who were not included in the main study. Internal consistency was measured using Cronbach’s alpha, yielding a value of 0.75. Test-retest reliability was evaluated using the intraclass correlation coefficient (ICC) and the weighted kappa coefficient, with values of approximately 0.66 and 0.30, respectively. Overall, the questionnaire items were found to be generally understandable, and the expert evaluations confirmed that the items effectively captured the intended constructs, thereby supporting the instrument’s validity and reliability. The questionnaire was distributed online via Google Forms as a link via phone message. A signed consent form was electronically endorsed by each participant. The online survey included a hyperlink to the consent form for the participants’ personal use. The consent forms were cross-referenced to verify participant identities, and the questionnaire form was designed to only allow one completion.

The questionnaire consisted of two sections and a total of 34 questions. The first section of the questionnaire included eight questions regarding the sociodemographic information of the healthcare participant. The second section of the questionnaire included 26 questions regarding drug administration via EFT and questions regarding common errors and mistakes detected in the OP. A precise and objective measurement of the healthcare team’s knowledge and performance was provided by giving 1 point for correct answers and 0 points for wrong answers. Accordingly, participants in the questionnaire were evaluated with a minimum of 0 points and a maximum of 26 points.

### Statistical analysis

The study used descriptive statistics to present the central tendency and variability of continuous variables, including mean, median, standard deviation, interquartile range (IQR), or count and percentages, as appropriate. For categorical variables, frequency and percentages were provided. The normality of continuous variables was evaluated using the Kolmogorov-Smirnov test, which was found to have a non-parametric distribution. Mann-Whitney U tests were utilised for continuous variables to examine differences among groups, while Chi-square tests were employed to investigate relationships between categorical variables. Internal consistency of the knowledge questions was assessed using Cronbach’s alpha, and test-retest reliability was evaluated with both weighted kappa coefficients and Intraclass Correlation Coefficient (ICC). McNemar’s test was employed to compare paired questionnaire responses obtained before and after the educational intervention. Statistical significance was set at a 95% confidence interval and p-value < 0.05. Missing data were excluded from the analysis, and the entire dataset was processed using IBM SPSS Statistics for Windows, Version 29.0 (Armonk, New York: IBM Corp.).

## Results

**FIGURE 2:**
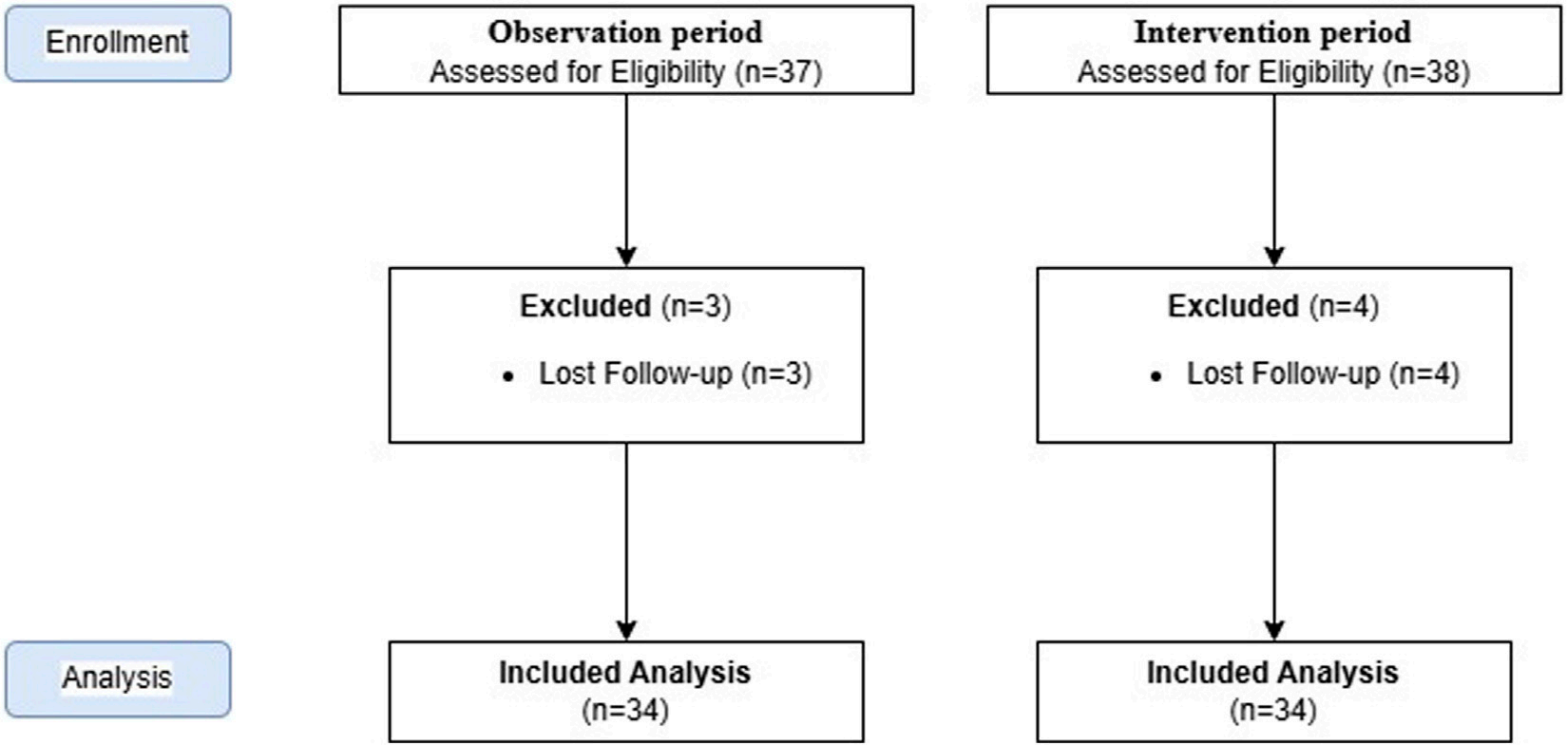
Study flowchart.

A total of 68 patients, 34 patients in OP and 34 patients in IP were included in the study (Figure 2). 44.1% and 61.8% of the patients were male in OP and IP, respectively. The median (IQR) Modified Rankin Scale (mRS) scores for patients in the OP and IP were 4 (1.75–4) and 1 (0–2.25), respectively (p < 0.001). (Table 1). In OP and IP, 76.5% and 88.2% of the patients were fed via nasogastric tube, and medications were administered this way (Table 2). During the OP, 47 drug administrations via the EFT were considered inappropriate for hospitalised patients. Appropriate dosage form alternatives were unavailable in the hospital for 51% (n = 24) of the drugs. EC and controlled-release (CR) tablets were used inappropriately for EFT in patients during their hospitalisation in both periods (Table 3). Table 4 presents the recommended alternatives for drugs that are frequently administered inappropriately via EFTs, including acetylsalicylic acid, pantoprazole, levetiracetam, metoprolol, nifedipine, trimetazidine, and donepezil.

**TABLE 1:** Sociodemographic data of the study participants (patients).

Variable	Observation period (n = 34)	Intervention period (n = 34)	*p*- value
Age (years), Median (IQR)	66.5 (58.5–77.25)	72 (55.5–82.5)	0.227
Sex, n (%)			0.224
Male	15 (44.1)	21 (61.8)	
Female	19 (55.9)	13 (38.2)	
BMI (kg/m^2^), Median (IQR)	23.8 (29.4–30)	25.7 (15.8–29.3)	0.699
Reason for hospitalization, n (%)			—
*Cerebrovascular accident*	25 (73.5)	31 (91.1)	
*Other*	9 (26.5)	3 (8.9)	
Comorbidities (ICD-10 code), n (%)			—
*Hypertension*	27 (79.4)	21 (61.7)	
*Diabetes mellitus*	18 (52.9)	13 (38.2)	
*Hyperlipidemia*	2 (5.8)	3 (8.8)	
*Alzheimer’s disease*	4 (11.7)	2 (5.8)	
*Chronic kidney disease*	8 (23.5)	1 (2.9)	
*Coronary artery disease*	4 (11.7)	1 (2.9)	
*Other*	25 (73.5)	24 (70.5)	
FOIS score (admission), Median (IQR)	2 (1–3.25)	2 (2–3.25)	0.286
FOIS score (day 90), Median (IQR)	4 (1.25–5)	5 (2–7)	0.370
Number of comorbidities, Median (IQR)	3 (2–3)	2 (1–3)	0.109
Total length of stay (day), Median (IQR)	13 (8.75–17)	14 (9.75–19)	0.274

BMI, body mass index; FOIS, Functional Oral Intake Scale; IQR, interquartile range; NIHSS, national institutes of health stroke scale.

**TABLE 2:** Findings of drug administration through enteral feeding tubes.

Variable	Observation period (n = 34)	Intervention period^a^ (n = 34)	*p*- value
Number of patients with inappropriate drug administration via EFT (during hospital stay), n (%)	24 (70.6)	13 (38.2)	0.014
Total number of inappropriate drug administrations via EFT (during hospital stay), Median (IQR)	1 (0–2)	0 (0–1)	<0.001
Number of patients with inappropriate medication administration via EFT (on discharge), n (%)	26 (76.5)	8 (23.5)	<0.001
Total number of inappropriate drug administrations from EFT (on discharge), Median (IQR)	1 (0.75–2.25)	0 (0–0.25)	<0.001
Number of patients experiencing EFT clogging, n (%)	6 (82.3)	3 (8.8)	0.476
Total number of EFT clogging, Mean (±SD)	0.47 ± 1.28	0.08 ± 0.28	0.235
EFT type, n (%)			
*Nasogastric tube*	26 (76.5)	30 (88.2)	0.340
*Percutaneous endoscopic gastrostomy*	8 (23.5)	4 (11.8)	
EFT usage period (days)	8 (5–12.25)	11 (7.75–18)	0.012
Total number of medications administered through EFT	6 (3–9)	7 (5–8)	0.509
Frequency of drug administration from EFT (daily)	6.5 (3.75–10.25)	8 (6.75–10)	0.257
Total number of prescriptions reviewed	12 (6.75–15.5)	12 (8.75–15.5)	0.431

EFT, enteral feeding tube; IQR, interquartile range.

^a^ The numbers indicate the values recorded before clinical pharmacist interventions were applied.

**TABLE 3:** Characteristics of drugs administered inappropriately through an enteral feeding tube during admission and discharge.

Variable	During hospital stay		Discharge	
	Observation group	Intervention group^a^	Observation group	Intervention group
Anatomical Therapeutic Chemical classification, n (%)				
*Digestıve system and metabolism*	15 (31.9)	1 (7.1)	16 (32.6)	4 (50)
*Blood and blood-formıng organs*	13 (27.6)	9 (64.2)	13 (26.5)	3 (37.5)
*Cardiovascular system*	12 (25.5)	1 (7.1)	8 (16.3)	0 (0)
*Other*	7 (14.8)	3 (21.6)	12 (26.5)	1 (12.5)
Active ingredient, n (%)				
*Acetylsalicylic acid*	13 (27.6)	9 (64.2)	13 (26.5)	3 (37.5)
*Pantoprazole*	8 (17)	0 (0)	16 (32.6)	3 (37.5)
*Levetiracetam*	5 (10.6)	0 (0)	0 (0)	0 (0)
*Metoprolol*	5 (10.6)	1 (7.1)	6 (12.2)	0 (0)
*Other*	16 (34.2)	4 (28.7)	14 (28.5)	2 (25)
Dosage form, n (%)				
*Enteric coated tablet*	22 (46.8)	9 (64.2)	30 (61.2)	6 (75)
*Controlled release tablet*	13 (27.6)	4 (28.7)	13 (26.5)	0 (0)
*Tablet*	7 (14.8)	0 (0)	2 (4)	1 (12.5)
*Gastroresistant tablet*	3 (6.3)	0 (0)	0 (0)	0 (0)
*Soft gelatin capsule*	2 (4.2)	1 (7.1)	4 (8.1)	1 (12.5)
Total	47 (100)	14 (100)	49 (100)	8 (100)

^a^ The numbers indicate the values recorded after clinical pharmacist interventions were applied.

**TABLE 4:** Commonly inappropriately administered drugs via enteral feeding tube and alternative recommendations.

Drug	Dosage form	Alternative recommendation
Acetylsalicylic Acid	Enteric coated tablet	Use non-enteric coated dosage forms
Pantoprazole	Enteric coated tablet	Use Lansoprazole micropellet capsule opened and prepared as a solution with 10 ml of 8.4% sodium bicarbonate
Levetiracetam	Film coated tablet	Use the syrup form
Metoprolol	Zero order kinetic release tablet	Use film-coated tablets that are not of controlled-release type
Nifedipine	Controlled release tablet	Use film-coated tablets that are not of controlled-release type
Trimetazidine	Modified released tablet	Use film-coated tablets that are not modified-release
Donepezil	Film coated tablet	Use the effervescent tablet form

Regarding the knowledge levels of the neurology team in EFT medication administration, 60% were nurses and 40% were physicians, with an average age of 27.6 ± 4.5 years. Sixty-eight percent of the participants had more than 10 years of experience in neurology. Fifty-two percent of the participants held an undergraduate degree, while the remaining 48% had postgraduate qualifications. Additionally, 64% indicated that they received less than 1 hour per week of education on drug preparation and administration (Table 5).

**TABLE 5:** Information of the healthcare team participating in the questionnaire.

Variable	n = 25
Participants, n (%)	
Neurologist	10 (40)
Nurse	15 (60)
Sex, n (%)	
Male	2 (8)
Female	23 (92)
Age, Mean (years) (±SD)	27.6 ± 4.5
Education level, n (%)	
Bachelor’s Degree	13 (52)
Master’s Degree	10 (40)
Doctorate	2 (8)
Experience in neurology (years), n (%)	
0–10	8 (32)
10–20	5 (20)
20–30	3 (12)
>30	9 (36)
How many patients do you provide daytime care to in a day?, Mean (±SD)	17.2 ± 13.6
How many patients do you care for at night in a day?, Mean (±SD)	13.8 ± 8.7
How many hours per week do you receive training on the preparation and administration of medications?	
Less than 1 h	16 (64)
10 and above	2 (8)
1–5 h	6 (24)
6–10 h	1 (4)

SD, standard deviation.

Following the educational intervention, a significant reduction in inappropriate medication administration was observed. The rate of inappropriate EFT medication administration during hospitalization decreased from 70.6% in OP to 38.2% in IP (p = 0.014) (Table 2). Similarly, at hospital discharge, the rate decreased from 76.5% in OP to 23.5% in IP (p < 0.001). Education also significantly improved the knowledge levels of the neurology team. The mean (±SD) correct answer score increased from 16.1 ± 4 to 21.1 ± 2.1 (p < 0.001). Specifically, improvements were observed in knowledge of the required fluid volume for flushing (question 5), the duration of feeding breaks (question 15), and the importance of avoiding modified-release drug manipulation (questions 20, 21, 22, and 23), as shown in Table 6.

**TABLE 6:** Questionnaire questions and distribution of correct answers by the healthcare team.

Variable	Pre-education	Post-education	*p*- value
Q1. Direct EFT application of liquid-form drugs is appropriate	0	3	0.250
Q2. To ensure EFT patency, fluid should be administered before drug administration	25	25	1.000
Q3. Dispensing in a ready-to-administer package prepared directly at the pharmacy or in an individualized drug supply system can reduce the percentage of medication errors	21	22	1.000
Q4. Procedures, posters, and brochures regarding medication administration from EFT inwards help reduce medication administration errors	22	25	0.250
Q5. The amount of liquid you use to flush the EFT after applying the medication is 30 ml or more	11	24	<0.001
Q6. Medicines to be administered through EFT are diluted with tap water	3	9	0.109
Q7. Shift changes (double shifts, overtime, etc.) and workload can lead to medication preparation errors	20	24	0.219
Q8. For the safe management of the preparation and administration process of drugs to be administered through EFT, some authoritative guidelines are required, taking into account the available scientific evidence	22	24	0.625
Q9. Clinical skills regarding the safe management of drug therapy should be assessed regularly	20	25	0.630
Q10. Protocols/guidelines/procedures improve professional behavior and ensure appropriate management of therapeutic processes	21	25	0.125
Q11. Organizing continuous and specific training on the safe administration of medications from EFT can reduce the risk of medication administration errors from EFT.	22	23	1.000
Q12. Educational materials to be placed in the field regarding drug preparation and administration prevent drug administration errors from EFT during the drug administration process	21	25	0.125
Q13. The enteral tube should be washed for interventions after drug administration	21	25	0.125
Q14. During simultaneous administration of more than one drug, the drugs can be combined and given in the same liquid	16	19	0.508
Q15. After applying the medication, the period without feeding should be at least 1 h	13	21	0.039
Q16. My knowledge of drug preparation and administration is sufficient	10	16	1.000
Q17. Regular training on drug preparation and administration should be organized to reduce medication errors	21	23	0.688
Q18. Educational materials on drug preparation and administration are useful for optimizing drug administration	25	24	1.000
Q19. I need a clinical pharmacist in the neurology field whom I can consult as needed	22	23	1.000
Q20. The drug tablet in zero-order kinetic release dosage form can be crushed and administered through an enteral tube	12	23	0.003
Q21. The tablet of a drug in a controlled-release dosage form can be administered through an enteral tube by crushing it in a mortar	8	22	0.001
Q22. The drug tablet in the extended-release dosage form can be administered through an enteral tube by crushing/breaking it in a mortar	8	21	<0.001
Q23. All medicines in capsule form can be administered by opening/crushing/diluting the capsule	12	20	0.039
Q24. When administered enterally, enteric-coated, controlled-release tablets or capsules are prepared by crushing and mixing in a mortar	11	15	0.481
Q25. Metoprolol zero-order kinetic tablet use is requested for a patient diagnosed with atrial fibrillation. Which of the following preparations is a more suitable agent to be administered through EFT instead of the active substance metoprolol? (bisoprolol tablet)	11	11	1.000
Q26. For the patient receiving dual antiplatelet therapy, proton pump inhibitors are requested to be administered via the nasogastric route. Which of the following preparations would be more appropriate to administer from EFT than other agents? (lansoprazole capsule)	5	12	0.092
Total score, Mean (±SD)	16.1 ± 4	21.1 ± 2.1	<0.001

EFT, enteral feeding tube; SD, standard deviation.

In IP, EFTRMAEs were detected in 13 patients before clinical pharmacist interventions. During the hospitalisation of these 13 patients in IP, of the 25 clinical pharmacists’ interventions regarding 25 inappropriate drug administrations via the EFT, 84% (n = 21) were accepted by the healthcare team. 11 of these recommendations were implemented, and the remaining inappropriate dosage forms continued to be used. Of the 21 recommendations provided by the clinical pharmacist in the IP, 4 recommendations were provided regarding an alternative pharmacological group with an appropriate dosage form, and 17 recommendations regarding a crushable/breakable dosage form of the same drug. Of the 14 drugs that caused inappropriate administration in IP 78.5% (n = 11) did not have an appropriate dosage form alternative in the hospital. Consequently, in the inpatient setting, inappropriate drug administration via EFT remained in only five patients (14.7%) (p < 0.001). A total of 8 inappropriate dosage forms from EFT were identified during the discharge process in IP. Since these dosage forms did not have alternatives in appropriate dosage and form outside the hospital, no recommendations were provided.

## Discussion

In this study, we investigated the effects of clinical pharmacy interventions and education programs on reducing EFTRMAEs in patients hospitalised in neurology wards. The findings from this study reported frequent inappropriate drug administration via EFT in neurology wards. In addition to increasing the knowledge level of healthcare staff, physicians’ practice of prescribing appropriate dosage forms via EFT was also improved. Thus, we observed that clinical pharmacists reduced EFTRMAEs rates by 45.8% and 69.2% during hospitalisation and discharge, respectively. The prevalence of EFTRMAEs presents a pressing challenge in patient management, particularly in intensive care units (ICUs) (Abreu et al., 2021; Walther et al., 2018; Yu et al., 2020)^.^ Previous studies found that EFTRMAEs were a common occurrence in hospitalised patients, with a frequency of 43%–80% (Buhrer Ferreira Neto et al., 2016; Lohmann et al., 2015; Walther et al., 2018), such as inappropriate formulations of extended-release or EC medications (Abreu et al., 2021; Yu et al., 2020). This highlights the urgent need for interventions aimed at reducing such DRPs. In the present study, there were 47 inappropriate drug administrations via the EFT detected in the OP. Furthermore, the high rate of unavailability of alternative dosage forms of drugs causing EFTRMAEs may have increased the error rates. This is a common occurrence, as reported in previous studies. Appropriate dosage form alternatives were unavailable in the hospital for 51% of the drug administration. EC and CR tablets were used inappropriately for EFT in patients during their hospitalisation. The continued inappropriate use of EC and CR tablets in the post-intervention phase can be attributed primarily to the limited availability of suitable alternative dosage forms in the hospital formulary, as evidenced by the fact that appropriate alternatives were unavailable in 51% of drug administrations. Additionally, deeply entrenched prescribing habits and a degree of resistance to change among clinicians may further contribute to the sustained use of these formulations despite the educational and clinical pharmacy interventions. Our findings were consistent with previous studies (Demirkan et al., 2017; Wasylewicz et al., 2021). In addition, the low rate of appropriate dosage forms not applied via EFT in the market and, therefore, the high rate of off-label use of drugs are among the factors that increase EFTRMAEs (Buhrer Ferreira Neto et al., 2016; Zuccari et al., 2022).

In this study, the dosage forms, active ingredients, and ATC classifications of the drugs that frequently cause EFTRMAEs are similar to those in the literature (Wasylewicz et al., 2021)^.^ Sommerfeldt et al. evaluated the drugs applied via EFT during the discharge process in a stroke unit and found that the most frequently applied drugs were stated as bisoprolol, candesartan and ramipril, acetylsalicylic acid, amlodipine, hydrochlorothiazide, omeprazole, and esomeprazole (Sommerfeldt et al., 2024).

The high prevalence of EFTRMAEs among hospitalized patients in our study, along with the observed reduction following educational interventions, the integration of clinical decision support systems, and clinical pharmacist involvement, is consistent with findings from previous studies conducted by Abreu et al. (2021); Dashti-Khavidaki et al. (2012); Idzinga et al. (2009); Lohmann et al. (2015); Walther et al. (2018); Yu et al. (2020). The findings of our study highlighted the essential role of clinical pharmacists interventions in reducing EFTRMAEs in hospital settings. While the education program improved healthcare professionals’ knowledge regarding EFT medication administration, the additional impact of clinical pharmacist interventions was observed in optimizing medication selection and prescribing practices. These interventions specifically addressed the lack of appropriate dosage forms, proposed alternative formulations, and ensured safer administration methods beyond the scope of the educational program. This distinction highlights that while education provided a foundation for understanding proper EFT medication administration, direct pharmacist involvement was necessary to implement and sustain improvements in clinical practice. These findings were in accordance with a study conducted by Walther et al. with pre- and post-education in the neurology ward and neurosurgery ICU to optimise medication administration and prescriptions via EFT during the admission and discharge of patients. The study found that inappropriate prescriptions via EFT were mainly related to modified-release medications. However, the frequency of inappropriate EFT decreased from 44% to 25% after pharmacist intervention (p < 0.05) (Walther et al., 2018). Similar findings were also reported in the study by Wasylewicz et al. conducted as a pre-post study with a decreased incidence of EFTRMAEs from 15% to 2% in the pre- and post-intervention period (p < 0.05) (Wasylewicz et al., 2021).

However, it should be noted that different EFTRMAE definitions and drug administration procedures have also been evaluated for EFT in previous studies. Considering this perspective, only inappropriate selection of drug dosage forms was evaluated as EFTRMAE in this study. In addition, while most of the studies focused on hospitalised patients, EFTRMAEs were also evaluated during patient discharge in our study. In line with earlier studies conducted by clinical pharmacists in the neurology ward, it was found that interventions aimed at reducing EFTRMAEs were highly accepted and implemented by the healthcare team and the frequency of errors was significantly reduced compared to patients in the OP (Liu et al., 2021)^.^ In this context, Zuccari et al. also conducted a study to evaluate the role of the pharmacist in selecting and administering appropriate drug formulations through EFT for dysphagic patients. Accordingly, 1047 solid oral pharmaceutical forms included in the hospital formulary were evaluated. The review highlighted that 95% of these drugs were used off-label, which is alarming, and 40% were used without appropriate indications from manufacturers or literature studies. The study also highlighted that hospital pharmacists should be involved to manage these DRPs (Zuccari et al., 2022).

On the other hand, the dynamic collaboration between pharmacists and nursing staff is emerging as an essential factor in promoting safe medication administration. The study of Ferreira Neto et al. found that many medications prescribed for EFT are either contraindicated or require more adequate information about their use. This knowledge gap among prescribers, combined with inadequate instructions provided on medication packaging, requires comprehensive intervention (Buhrer Ferreira Neto et al., 2016). Overcoming these challenges requires the establishment of multidisciplinary teams. Standardising safe medication administration techniques and providing educational sessions for healthcare practitioners will also be crucial to creating a culture of safety and compliance.

### Impact of the education program on the knowledge level of the healthcare team for drug administration via EFT

In this study, the knowledge level of the healthcare team about drug administration via EFT was also evaluated before and after pharmacist education and intervention. It was determined that the healthcare team participating in the education program was primarily experienced in the neurology ward. However, the frequency of education about drug administration via EFT was quite low. Alhashemi et al. reported that half of the nurses chose pharmacists as the first source to consult regarding medication administration (Alhashemi et al., 2019). Although drug administration procedures are primarily nurses part, physicians also need to be included in the education programs in order to properly select the drug dosage form and reduce the rate of EFTRMAEs (Mota et al., 2010). Therefore, in our study, physicians and nurses in the neurology ward were included in the educational program provided by clinical pharmacists about drug administration via EFT. In particular, the widespread use of CR, EC tablets and other dosage forms that are inappropriate for administration via EFT, which cause EFTRMAEs due to their manipulations, was reduced in IP with the increase in knowledge level after education and the recommendations of the clinical pharmacist. Moreover, after the educational program in IP, the high rate of acceptance of the recommendations of clinical pharmacists regarding medication administration by physicians and the increase in the consultation rates of nurses resulted in the successful reduction of EFTRMAEs.

These findings were consistent with earlier studies that showed an integrated intervention program focused on encouraging the correct administration of medications by clinical pharmacists through EFT significantly increased the knowledge of nurses, especially in terms of drug preparation, tube flushing, recognising drug-drug/drug-food interactions, and recognising the characteristics of dosage forms (Van Den Bemt et al., 2006; Hanssens et al., 2006).

### Strengths and limitations

This study is among the few in the literature that provides evidence supporting the reduction of EFTRMAEs through clinical pharmacist interventions and educational initiatives. In comparison to other studies, education on drug administration using EFT was provided to physicians and nurses. Reflecting on the positive results of education and clinical pharmacy services from EFT to drug administration was one of the strengths of this study. One of the study’s limitations is that it was conducted in a single centre with a small number of patients. We emphasise that the study cannot be generalised due to differences in the knowledge level of those who administer and prescribe medication via the EFT. In future studies, randomised controlled studies involving multiple centres will further highlight the role of the clinical pharmacist in this field. Furthermore, future research that adopts a more comprehensive approach to clinical outcomes may further elucidate the potential relationship between improved medication administration practices and hospital length of stay. This broader perspective would allow for a more holistic evaluation of not only patient safety but also the overall efficiency and resource utilization of healthcare institutions.

## Conclusion

While the current study highlights a troubling landscape of EFTRMAEs, it also offers a promising avenue for improvement through targeted education, pharmaceutical interventions, and improved collaboration between pharmacists and healthcare professionals. By prioritising accuracy in medication prescribing and fostering an environment of continuous learning, healthcare organisations can significantly reduce the frequency of medication errors and ultimately improve patient safety and care outcomes.

## Data Availability

The raw data supporting the conclusions of this article will be made available by the corresponding author upon reasonable request.

## References

[B1] Abreu G. A. Chaves E. F. Neto J. A. Batista M. M. Costa L. S. Silva R. P. (2021). Off-label use of drugs administered by enteral feeding tubes in an Intensive Care Unit in Fortaleza, Brazil. Rev. Bras. Farmácia Hosp. Serv. Saúde 12 (1), 562. 10.30968/rbfhss.2021.121.0562

[B2] Abu Hdaib N. Albsoul-Younes A. Wazaify M. (2021). Oral medications administration through enteral feeding tube: clinical pharmacist-led educational intervention to improve knowledge of Intensive care units’ nurses at Jordan University Hospital. Saudi Pharm. J. 29 (2), 134–142. 10.1016/j.jsps.2020.12.015 33679176 PMC7910138

[B3] Alhashemi S. H. Ghorbani R. Vazin A. (2019). Improving knowledge, attitudes, and practice of nurses in medication administration through enteral feeding tubes by clinical pharmacists: a case-control study. Adv. Med. Educ. Pract. 10, 493–500. 10.2147/AMEP.S203680 31372085 PMC6628606

[B4] Al-Taie A. (2023). Implications of health care providers by physicians’ and pharmacists’ attitudes and perceptive barriers towards interprofessional collaborative practices. Braz. J. Pharm. Sci. 58, e20983. 10.1590/s2175-97902022e20983

[B5] Bankhead R. Boullata J. Brantley S. Corkins M. Guenter P. Krenitsky J. (2009). Enteral nutrition practice recommendations. J. Parenter. Enter. Nutr. 33 (2), 122–167. 10.1177/0148607108330314 19171692

[B6] Buhrer Ferreira Neto C. J. Plodek C. K. Soares F. K. Santos M. A. Lima T. R. Nogueira C. S. (2016). Pharmaceutical interventions in medications prescribed for administration via enteral tubes in a teaching hospital. Rev. Lat. Am. Enferm. 24, e2696. 10.1590/1518-8345.0619.2696 PMC4915797 27276019

[B7] Cengiz K. N. Midi I. Sancar M. (2025). The effect of clinical pharmacist-led pharmaceutical care services on medication adherence, clinical outcomes, and quality of life in patients with stroke: a randomised controlled trial. Int. J. Clin. Pharm. 47 (1), 99–106. 10.1007/s11096-024-01811-0 39395139

[B8] Coppens C. H. van den Engel-Hoek L. Scharbatke H. Broekhuizen J. L. Derks M. A. Janssen B. A. (2016). Dysphagia in children with repaired oesophageal atresia. Eur. J. Pediatr. 175 (9), 1209–1217. 10.1007/s00431-016-2760-4 27544282 PMC5005404

[B9] Crary M. A. Carnaby Mann G. D. Groher M. E. (2005). Initial psychometric assessment of a functional oral intake scale for dysphagia in stroke patients. Arch. Phys. Med. Rehabil. 86 (8), 1516–1520. 10.1016/j.apmr.2004.11.049 16084801

[B10] Dashti-Khavidaki S. Badri S. Eftekharzadeh S. Z. Rahmani R. Zargar A. Ahmadi M. (2012). The role of clinical pharmacist to improve medication administration through enteral feeding tubes by nurses. Int. J. Clin. Pharm. 34 (5), 757–764. 10.1007/s11096-012-9673-8 22790463

[B11] Demirkan K. Bayraktar-Ekincioglu A. Gulhan-Halil M. Keskin A. S. Basaran S. Dincer E. (2017). Assessment of drug administration via feeding tube and the knowledge of health-care professionals in a university hospital. Eur. J. Clin. Nutr. 71 (2), 164–168. 10.1038/ejcn.2016.147 27507069

[B12] Hanssens Y. Woods D. Alsulaiti A. Singh D. Ahmed M. Hassan R. (2006). Improving oral medicine administration in patients with swallowing problems and feeding tubes. Ann. Pharmacother. 40 (12), 2142–2147. 10.1345/aph.1H342 17132805

[B13] Idzinga J. C. De Jong A. L. Van den Bemt P. M. L. A. (2009). The effect of an intervention aimed at reducing errors when administering medication through enteral feeding tubes in an institution for individuals with intellectual disability. J. Intellect. Disabil. Res. 53 (11), 932–938. 10.1111/j.1365-2788.2009.01212.x 19744260

[B14] Liu P. Li G. Han M. Zhang L. Wang Y. Huang J. (2021). Identification and solution of drug-related problems in the neurology unit of a tertiary hospital in China. BMC Pharmacol. Toxicol. 22 (1), 65–69. 10.1186/s40360-021-00530-w 34702348 PMC8547903

[B15] Lohmann K. Gartner D. Kurze R. Schmidt P. Weber M. Haas S. (2015). More than just crushing: a prospective pre-post intervention study to reduce drug preparation errors in patients with feeding tubes. J. Clin. Pharm. Ther. 40 (2), 220–225. 10.1111/jcpt.12250 25655434

[B16] Mota M. L. S. Barbosa I. V. Studart R. M. B. Costa J. L. Lima F. M. Nunes M. S. (2010). Evaluation of intensivist-nurses’ knowledge concerning medication administration through nasogastric and enteral tubes. Rev. Lat. Am. Enferm. 18 (5), 888–894. 10.1590/s0104-11692010000500008 21120407

[B17] Nieuwenhuizen W. F. Weenen H. Rigby P. Heijnen P. Geurts J. Kroos T. (2010). Older adults and patients in need of nutritional support: review of current treatment options and factors influencing nutritional intake. Clin. Nutr. 29 (2), 160–169. 10.1016/j.clnu.2009.09.003 19828215

[B18] Sheard J. M. (2014). Malnutrition and neurodegenerative diseases. Curr. Nutr. Rep. 3 (2), 102–109. 10.1007/s13668-014-0078-2

[B19] Sommerfeldt J. Sartorius H. von Sarnowski B. Schröder T. Kuhn P. Müller R. (2024). Drug administration via feeding tubes - a procedure that carries risks: systematic identification of critical factors based on commonly administered drugs in a cohort of stroke patients. Eur. J. Clin. Pharmacol. 80 (11), 1599–1623. 10.1007/s00228-024-03723-4 39073438 PMC11458809

[B20] Stavroulakis T. McDermott C. J. (2016). Enteral feeding in neurological disorders. Pract. Neurol. 16 (5), 352–361. 10.1136/practneurol-2016-001408 27152026

[B21] Van Den Bemt P. M. L. A. Cusell M. B. I. Overbeeke P. W. van Roon M. L. van den Broek P. H. Visser L. J. (2006). Quality improvement of oral medication administration in patients with enteral feeding tubes. Qual. Saf. Health Care 15 (1), 44–47. 10.1136/qshc.2004.013524 16456209 PMC2563995

[B22] Van Welie S. Wijma L. Beerden T. Hendriks R. van Vliet P. Koster T. (2016). Effect of warning symbols in combination with education on the frequency of erroneously crushing medication in nursing homes: an uncontrolled before and after study. BMJ Open 6 (8), e012286. 10.1136/bmjopen-2016-012286 PMC4985836 27496242

[B23] Walther J. Nivoix Y. Vigouroux D. Brun L. Forestier A. Klein C. (2018). Improvement of drugs prescription and administration through enteral feeding tubes during hospitalization and before discharge to home. Nutr. Clin. Metab. 32 (2), 113–121. 10.1016/j.nupar.2017.12.002

[B24] Wasylewicz A. T. M. van Grinsven R. J. B. Bikker J. M. W. van Dijk H. L. Kooijman S. R. van Wijk J. T. (2021). Clinical decision support system-assisted pharmacy intervention reduces feeding tube–related medication errors in hospitalized patients: a focus on medication suitable for feeding-tube administration. J. Parenter. Enter. Nutr. 45 (3), 625–632. 10.1002/jpen.1869 PMC8048796 32384187

[B25] White R. Bradnam V. (2018). Handbook of drug administration via enteral feeding tubes. 3rd ed. London: Pharmaceutical Press.

[B26] Williams N. T. (2008). Medication administration through enteral feeding tubes. Am. J. Health Pharm. 65 (24), 2347–2357. 10.2146/ajhp080155 19052281

[B27] Yu M. Chen J. Zheng S. Zhang L. Zhou Y. Wang T. (2020). Reduce medication errors in tube feeding administration by establishing administration standards and standardizing operation procedures. Drugs Ther. Perspect. 36 (2), 69–74. 10.1007/s40267-019-00698-6

[B28] Zahn J. Meißner T. Daniels R. Schneider H. Bergmann M. Fischer A. (2020). Manipulation of medicinal products for oral administration to paediatric patients at a German university hospital: an observational study. Pharmaceutics 12 (6), 1–16. 10.3390/pharmaceutics12060583 PMC7355957 32586000

[B29] Zuccari G. Macis S. Alfei S. Rossini R. Gervasoni M. Benelli P. (2022). The role of the pharmacist in selecting the best choice of medication formulation in dysphagic patients. J. Pers. Med. 12 (8), 1307. 10.3390/jpm12081307 36013259 PMC9410388

